# SARS-CoV-2 antibody responses before and after a third dose of the BNT162b2 vaccine in Italian healthcare workers aged ≤60 years: One year of surveillance

**DOI:** 10.3389/fimmu.2022.947187

**Published:** 2022-09-30

**Authors:** Monica Franzese, Luigi Coppola, Romina Silva, Stefano Angelo Santini, Luigi Cinquanta, Cosimo Ottomano, Marco Salvatore, Mariarosaria Incoronato

**Affiliations:** ^1^ Research Laboratory, Istituto di Ricerca e Cura a Carattere Scientifico (IRCCS) Synlab SDN Spa, Naples, Italy; ^2^ Laboratory of Medicine, Synlab Lazio Srl, Rome, Italy

**Keywords:** SARS-CoV-2, BNT162b2, vaccine, humoral response, booster, neutralizing antibodies

## Abstract

This study monitored the anti-spike-receptor-binding domain (RBD) and neutralizing antibodies induced by the Pfizer/BioNTech mRNA BNT162b2 vaccine in a cohort of 163 healthcare workers aged ≤60 years. We have taken advantage of two study groups, both of whom received the first two doses in the same time window, but Group 1 (54 HCWs) received the third dose 2 months before Group 2 (68 HCWs) did. The cohorts were monitored from the 12th day after the first vaccine dose up to 1 month after the third vaccine dose for a total of eight time points and about 1 year of surveillance (T1 = 12 days after the first dose; T2 = 10 days after the second dose; T3 = 1 month after the second dose; T4 = 3 months after the second dose; T5 = 4 months after the second dose; T6 = 5 months after the second dose; T7 = 7 months after the second dose; T8 = 1 month after the third dose for Group 1; T8* = 9 months after the second dose for Group 2; T9 = 1 month after the third dose for Group 2). The mean value of anti-spike antibodies decreased faster over time, but at T7, its decline was significantly slowed (T7 vs. T8*). After the third dose, the anti-spike titer rose about 34-fold (T7 vs. T8 and T8* vs. T9) and the booster improved the anti-spike titer by about three times compared with that of the second dose (T3 vs. T8 and T3 vs. T9), and no difference was noted between the two groups. The neutralizing titer was evaluated at T3, T7, T8, and T9. Anti-spike and neutralizing antibodies were found to be strongly correlated (r^2^ = 0.980; p < 0.001). At T3, 70% of the participants had a neutralizing antibody titer >91% of total anti-spike antibodies that increased to 90% after the third dose (T8 and T9). However, when the anti-spike titer reached its lowest value (T7), the neutralizing antibody levels decreased even further, representing only 44% of total anti-spike antibodies (p < 0.0001). Our findings show that the third vaccine dose improves the humoral response, but the wane of the anti-spike and neutralizing antibody titers over time is more marked in the neutralizing antibodies.

## Introduction

A new severe acute respiratory syndrome coronavirus 2 (SARS-CoV-2) was identified in December 2019 as the causative agent of pneumonia, and on 11 March 2020, the World Health Organization (WHO) declared a pandemic status. With a continuously and rapidly growing number of cases and deaths globally, an urgent need arose to identify therapies and develop a vaccine to counter the spread of the virus. The first vaccine to pass phase III clinical trials was Comirnaty (BNT162b2), developed by Pfizer/BioNTech. With a 95% efficiency rate achieved with two doses administered 21 days apart and with few side effects (1, 2), the vaccine was quickly approved.

Although BNT162b2 is highly efficacious against coronavirus disease 2019 (COVID-19) (3), decreased efficacy of the two vaccine doses over time has been demonstrated (4, 5). Following the increase in infection and hospitalizations several months after the second dose, the Israeli Ministry of Health approved a third SARS-CoV-2 BNT162b2 vaccine dose (booster). Its usefulness in terms of a decrease in infections (6) and reactogenicity (7) was clearly demonstrated. Therefore, on 4 October 2021, the European Medicines Agency (EMA) also recommended the third dose (https://www.ema.europa.eu/en/news/comirnaty-spikevax-ema-recommendations-extra-doses-boosters).

Reliable published data to date show that of the total immunoglobulin class G (IgG) anti-spike antibodies produced after vaccination or COVID-19 infection, antibodies against the receptor-binding domain (RBD) of S protein [neutralizing antibodies (nAbs)] are associated with immune protection against COVID-19 infection (8–10). In fact, such nAbs produced by COVID-19 patients can block viral infection of human cells *in vitro* and counter viral replication *in vivo* (11, 12). In addition, the lack of a nAb response predicts fatal outcomes (13). Kinetic nAb responses at 3 months (14) and 6 months (15) after the second dose were reported in a few studies, and sometimes the results were discordant (16).

The first study evaluating the humoral response before and after the third dose of the BNT162b2 vaccine was published in December 2021 (17). This study monitored only IgG anti-spike antibody titer in a cohort of individuals aged ≥60 years as a category at risk of hospitalization due to infection. Nevertheless, it is necessary also to know the trend of humoral responses before and after the third BNT162b2 dose in individuals ≤60 years, since this younger population also underwent the third dose to reduce the risk of hospitalization following infection.

Since the BNT162b2 vaccine immunity wanes over time and the need for a third dose has been shown, several questions remain unanswered: 1) How long do nAbs persist in the serum of subjects with two vaccine doses? 2) Is the trend in the wane of the nAbs similar to that of the anti-spike antibodies in the same time window? 3) Is the humoral response after the third dose similar to that after the second dose?

This study aimed to answer these questions by monitoring a cohort of 163 healthcare workers (HCWs) ≤60 years old who were vaccinated with the BNT162b2 mRNA vaccine. The sero-surveillance program was initiated at IRCCS Synlab SDN starting in January 2021. Our study took advantage of two study groups monitored up to 1 month after the third dose for 1 year of surveillance. Group 1 received the third dose in October 2021, and Group 2 received the third dose 2 months later; this allowed us to perform comparative analyses.

## Materials and methods

### Participants, blood collection and study design

This prospective study was approved by the IRCCS Pascale, Institutional Ethical Review Board (CE: Protocollo n. 4/21, 2021). The study cohort included HCWs (medical doctors, nurses, technicians, biologists) and nonmedical personnel of the IRCCS Synlab SDN Institute tested for their humoral adaptive immune response to the BNT162b2 mRNA COVID-19 (Comirnaty) vaccine. This study enrolled 186 HCWs who received two doses of the BNT162b2 vaccine (with 21-day intervals between the two doses) during January and February 2021. All participants provided informed consent. Exclusion criteria: 1) previous COVID-19 infection; 2) >60 years old; 3) pregnancy. A total of 163 HCWs were eligible for the study. Among them, 54 HCWs (33.1%) received the third dose (booster) in October 2021 (Group 1) and 68 HCWs (41.7%) received it 53 ± 9 days later (Group 2). The remaining 41 eligible participants were evaluated only up to pre-booster because, for them, not all of the time point measurements were available to perform matched-pair design pre- vs. post-booster.

**Figure 1:**
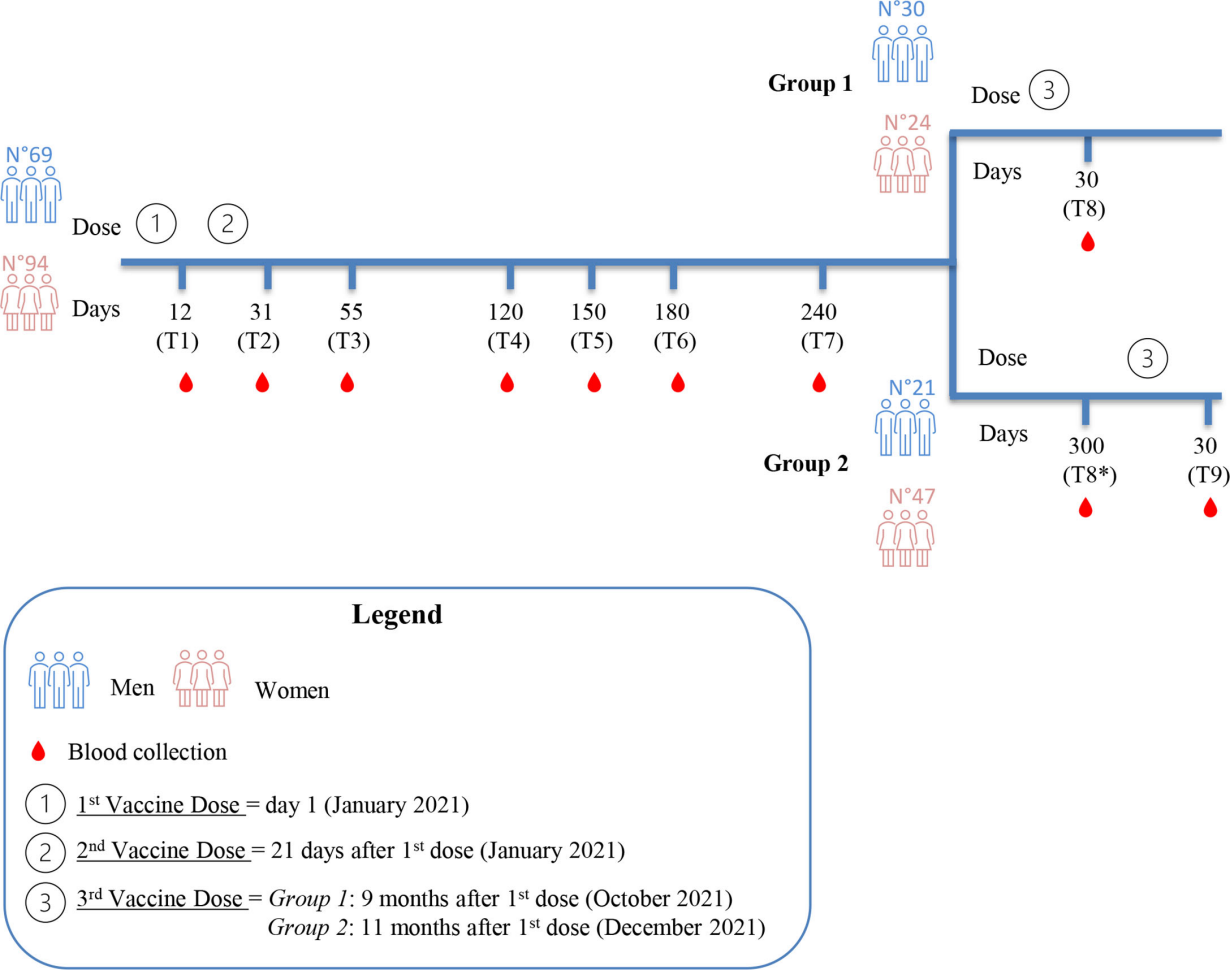
Study design. Blood collection at the indicated time points (T1 to T7) refers to the daysafter the first vaccine dose. After 240 days from the first dose, the study cohort was divided intotwo groups. Group 1 underwent the third dose 9 months after the first dose. Time points of Group1 are T1, T2, T3, T4, T5, T6, T7 and T8 (30 days after the third dose). Group 2 underwent the thirddose 11 months after the first dose, two months later than Group 1. Time points of Group 2 are T1,T2, T3, T4, T5, T6, T7, T8* (300 days after the first dose) and T9 (30 days after the third dose).

BD Vacutainer serum separation venous blood collection tubes (BD, Franklin Lakes, NJ, USA), containing whole blood, were centrifuged at 3,400 rpm for 10 min. After centrifugation, the serum was collected, aliquoted, and stored at -80°C at the SDN Biobank (18) for programmed serological evaluations. The time points for blood collection were the following: time 1 (T1), 12 days after the first dose; time 2 (T2), 31 ± 1 day after the first dose (corresponding to 10 ± 1 day after the second dose); time 3 (T3), 55 ± 4 days after the first dose (corresponding to 1 month ± 4 days after the second dose); time 4 (T4), 120 ± 5 days after the first dose (corresponding to 3.2 months ± 5 days after the second dose); time 5 (T5), 150 ± 4 days after the first dose (corresponding to 4.2 months ± 4 days after the second dose); time 6 (T6), 180 ± 6 days after the first dose (corresponding to 5.2 months ± 6 days after the second dose); time 7 (T7), 240 ± 7 days after the first dose (corresponding to 7.2 months ± 7 days after the second dose); time 8 (T8), 30 ± 4 days after the third dose (Group 1); time 8* (T8*), 300 ± 8 days after the first dose, corresponding to 9.1 months ± 8 days after the second dose (Group 2); and time 9 (T9), 30 ± 2 days after the third dose (Group 2). The study design is reported in **Figure 1**.

### Anti-nucleocapsid antibody detection

The SARS-CoV-2 IgG assay (Abbott; REF H14806R01) is an *in vitro* diagnostic (IVD) chemiluminescent microparticle immunoassay (CMIA) on the ARCHITECT i1000SR platform. This assay is designed to detect IgG antibodies against the nucleocapsid protein of SARS-CoV-2, as indicated in the manufacturer’s package insert. It can use both serum and plasma from individuals who may have been infected or suspected of having coronavirus disease. Interpretation of results: the cutoff is 1.4 Index Sample/Control (S/C); when it is <1.4, it is negative, and when it is ≥1.4, it is positive. This test was performed to exclude from the cohort any subject who had previously been infected with SARS-CoV-2. To this end, at the T1, the serum samples of the enrolled subjects (N = 186) were assayed for IgG anti-nucleocapsid protein.

### Receptor Binding Domain (RBD)-specific antibody detection

The SARS-CoV-2 IgG II Quant assay (Abbott; REF H13918R01) is an IVD-CMIA method. Following the manufacturer’s package insert, this assay determines IgG antibodies to SARS-CoV-2 in human serum and plasma on the ARCHITECT i1000SR platform. This assay detects the IgG anti-spike-RBD response after the vaccine (Pfizer-BioNTech) and SARS-CoV-2 infection. The cutoff is 7 BAU WHO/ml; when it is <7, it is negative, and when it is ≥7.1, it is positive. Serum samples of all of the time points (T1, T2, T3, T4, T5, T6, T7, T8, T8*, and T9) were assayed for the presence of IgG against spike RBD.

### SARS-CoV-2 neutralizing antibody detection

The SARS-CoV-2 nAb kit (SGM Italy; REF 8003) is an immunoturbidimetric latex method (PETIA) kindly provided by Abbott. This assay is a surrogate virus neutralization test certified as a CE-IVD device under the *In Vitro* Diagnostic Directive (IVDD 98/79/EC) and used to quantify *in vitro* the serum nAbs that bind specifically to the binding domain of the RBD receptor [interaction site of RBD and human Angiotensin Converting Enzyme-2 (ACE2) receptor cell]. The automated assay nAb, on platform ABBOTT ALINITY C, uses the principle of antigen–antibody reaction and competition between antigens. Therefore, the process in which nAbs, by binding the RBD protein, block the interaction between RBD and ACE2 is simulated *in vitro*. Thus, the higher the nAb concentration, the weaker the reaction between ACE2 antigen and RBD. The higher the nAb concentration, the lower the absorbance value found and, consequently, the higher the inhibition rate. Measurement range: Linear nAb interval 0%–100% nAb%. According to the manufacturer’s package insert, the results can be interpreted as follows: the reference value, intended as the cutoff value, corresponds to 25nAb% of inhibition, a value between 25% and 56% indicates a low to moderate inhibition, whereas a value >56% indicates a high inhibition. The serum samples collected at the time points T3, T7, T8, and T9 were assayed for the presence of nAbs.

### Statistical analyses

The prior power analysis to calculate the minimum sample size was performed using G*Power software (version 3.1.9.2) to compare multiple groups associated at the time points. Statistical analysis was performed using the R Core Team (version 4.0.0, Austria). Continuous variables were expressed as mean and standard deviation (SD). Data distribution was tested for normality through the Shapiro–Wilk test, and Wilcoxon rank-sum test was used to compare pair groups based on a non-normal distribution. A p-value <0.05 was considered significant, and Bonferroni’s correction was used for multiple hypothesis corrections. A Spearman’s correlation analysis was run to investigate whether there was an association among variables in two groups separately. Spearman’s ρ >0.8 and significant p-value (p < 0.05) were set as thresholds to identify strong agreement between variables.

## Results

### Serological evaluation of SARS-CoV-2 nucleocapsid antibodies

To calculate the minimum sample size for the study design, we performed *a priori* power analysis using the ANOVA test to compare eight groups associated with different time points with the following assumptions: the power of about 0.8, an alpha level of 0.05, and effect size (f = 0.4). Based on the assumptions, the required minimum total sample size was 111.

This study enrolled 186 HCWs (median age 42 years) who received three doses of the BNT162b2 mRNA vaccine. Of these, 14 HCWs were excluded because they were over 60 years. The remaining 172 HCWs were screened for anti-nucleocapsid antibodies, a marker of previous SARS-CoV-2 infection. Among them, nine were excluded because at T1, they were positive for the presence of anti-nucleocapsid antibody (>1.4 U/ml) (data not shown). The remaining 163 HCWs aged ≤60 years and with undetectable serum SARS-CoV-2 anti-nucleocapsid antibodies were eligible for the study. The cohort underwent weekly nasopharyngeal swab tests throughout the study to monitor the presence of COVID-19 infection and according to Good Laboratory Practice (19).

### Kinetics of anti-spike-RBD antibodies before and after the third vaccination

**Figure 2:**
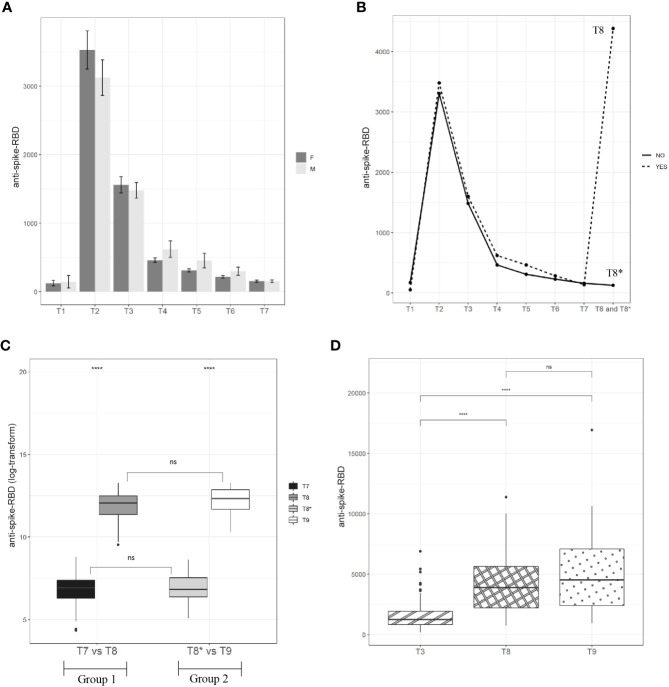
Kinetic of anti-spike-RBD antibodies. **(A)** A total of 94 vaccinated women and 69 men have been screened for anti-spike-RBD antibodies at the indicated time points up to 7.2 months after the second dose (T7). **(B)** A cohort of 163 vaccinated participants have been screened for anti-spike-RBD antibodies up to one month after the third dose (T8 for Group 1) and 9.1 months after the second dose (T8* for Group 2). **(C)** A total of 71 vaccinated participants (Group 1=54 and Group 2=68) have been screened for anti-spike-RBD antibodies before (T7 for Group 1 and T8* for Group 2) and after the third dose (T8 for Group 1 and T9 for Group 2). **(D)** Anti-spike-RBD antibodies mean value one month after the second dose (T3), one month after the third dose (T8, Group I) and one month after the third dose (T9, Group 2). Statistical significance was indicated by the star symbols (i.e., ns: p > 0.05, *p ≤ 0.05, ****p ≤ 0.000).

A total of 163 HCWs aged ≤60 years (women/men ratio was 94/69) were screened for the presence of anti-spike-RBD antibodies starting from 12 days after the first vaccine dose (T1) up to 7 months from the second dose of the vaccine (between T2 and T7) (see Materials and Methods and **Figure 1**). As reported in **Figure 2A**, the distribution of antibody titer values at the different time points was plotted by gender, but no significant differences were found (**Table S1**). Nevertheless, according to Notarte et al. (20, 21), we divided our cohort into <40 years and >40 years to compare the results by gender. As shown in **Figure S1A**, only in HCWs younger than 40 years was the anti-spike-RBD antibody titer at T3 and T4 significantly higher in women. No significant differences were found in HCWs >40 years (**Figure S1B**). At T1 (**Figure 2A** we found that 13.3% of HCWs (14 women and eight men) showed no humoral response, whereas the remaining 86.7% showed a mean value of anti-spike-RBD antibodies equal to 132.6 BAU/ml (women mean = 124.7 BAU/ml and men mean = 145.1 BAU/ml; **Table S2**). As reported in **Figure 2A**, at T2, we found the maximum humoral response: 100% of HCWs were positive for the presence of anti-spike-RBD antibodies, and the mean value rose to 25.4-fold compared with T1, reaching a value equal to 3,366.2 BAU/ml (women mean = 3,528.5 BAU/ml and men mean = 3,125.5 BAU/ml; **Table S2**). At 1 month after the second dose (T3), the mean anti-spike-RBD antibody value (1,526.5 BAU/ml) was half that at time T2, and at 3.2 months after the second dose (T4), the anti-spike-RBD antibody titer (525.2 BAU/ml) had dropped 6-fold compared with T2 (**Figure 2A**; **Table S2**). As reported in **Table S2**, the rapid decrease of anti-spike-RBD antibodies within the first 3 months after the second dose started to slow from time T5 onward (369.5 BAU/ml) up to T6 (253.9 BAU/ml) and T7 (153.2 BAU/ml). The anti-spike-RBD antibody decrease was statistically significant for each time point vs. each other (**Table S3**); and at T7, the anti-spike-RBD antibody amount reached values near those of T1 (**Table S2** and **Figure 2**).

While the antibody monitoring was proceeding in our cohort, the EMA approved the third dose (booster), and after about 9 months (October 2021) from the first vaccine dose (January 2021), we divided the cohort into two study groups because the enrolled HCWs underwent the third dose at separate times. Group 1, including 54 HCWs, immediately took the third dose (October 2021), while Group 2, including 68 HCWs, postponed the third dose by about 2 months (see *Materials and Methods M*&*M* and **Figure 1**). The division of the cohort into two groups allowed us to compare the anti-spike-RBD titers in the presence and absence of the booster dose. Specifically, as illustrated in **Figure 1**, the anti-spike-RBD antibody screening for Group 1 was performed 30 days after the third dose (T8), but for Group 2, this time point was 300 days after the first dose (or 9.1 months after the second dose, T8*); so, Group 2, in this time window, had not yet received the booster. As reported in **Figure 2B** and **Table S2**, after the booster, the anti-spike-RBD antibody titer of Group 1 increased by 34 times compared with that of Group 2 (T8 vs. T8*).

Interestingly, in the matched-pairs design, correlating humoral results at 7 (T7 = 129.7 BAU/ml) and 9 (T8* = 150.7 BAU/ml) months after the second dose, we did not see any significant difference (**Figure 2C**; **Tables S4**, **S5**). This result could mean that the wane in the anti-spike-RBD antibody titer had slowed starting from the seventh month after the second vaccine dose. But this is only a hypothesis because our study could not go beyond 9 months from the second vaccine administration. Then, we wondered whether the anti-spike-RBD antibody response after the third dose was similar to that after the second dose. To answer this, anti-spike-RBD antibody titers 1 month after the third dose (T8) and 1 month after the second dose (T3) were compared. **Figure 2D** and **Table S2** show that the anti-spike-RBD antibody titer after the third dose was 2.9 times greater than that of the second dose (T8 = 4,385.7 BAU/ml and T3 = 1,526.5 BAU/ml), highlighting the fact that the booster improved the humoral response. About 2 months later than Group 1, Group 2 received the third dose, and the anti-spike-RBD antibody mean titers at T3 and 1 month after the third dose (T9) were compared. As reported in **Figure 2D** and **Table S2**, the anti-spike-RBD antibody titer at T9 (5,073.8 BAU/ml) increased 3.3 times in comparison with T3 (1,526.5 BAU/ml). To assess whether the response to the booster dose between the two groups was the same, we compared the anti-spike-RBD antibody titers at T8 (Group 1) with those at T9 (Group 2), and no statistically significant differences were found (**Figures 2C, D**; **Table S4**). In addition, to assess if the antibody response was dependent on age before and after the booster dose, we stratified the two cohorts as <40 and ≥40 years, but no significant differences were found (data not shown). Taken together, these results showed that 1) from 7 months after the second dose, the decline in the anti-spike-RBD antibody titer appears to be significantly slowed; 2) the booster dose induces a much greater humoral response, improving it by about three times compared to the second dose; 3) after the third dose, there was no difference in the humoral response between Group 1 and Group 2, even though the booster was administered to Group 2 about 2 months later than that in Group 1.

### Relationship between anti-spike-RBD and neutralizing antibodies before and after the third vaccination

**Figure 3:**
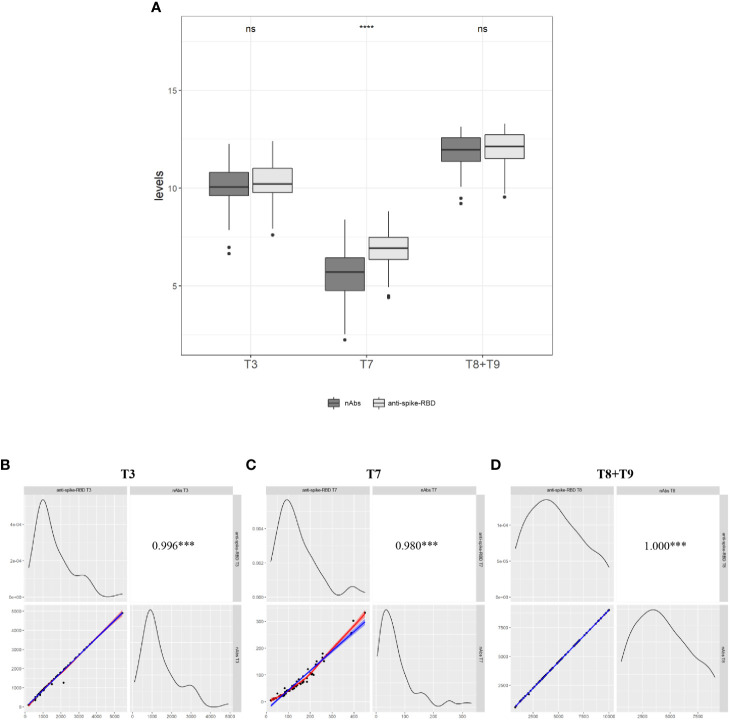
Relationship between anti-spike-RBD and anti-nAbs **(A)** The evaluation of both antibodies was conducted on a total of 71 participants (Group 1=41 and Group 2= 30) one month after the second dose (T3), 7.2 months after the second dose (T7) and one month after the third dose (T8 for Group and T9 for Group 2. Data merging = T+T9). **(B)** Correlation between anti-spike-RBD and anti-nAbs at T3, **(C)** at T7, and **(D)** at T8+T9. Statistical significance was indicated by the star symbols (i.e., ns: p > 0.05, ***p ≤ 0.001, ****p ≤ 0.000).

It is known that among the totality of IgG anti-spike-RBD produced after a vaccine or COVID-19 infection, antibodies against the RBD of the spike protein (nAbs) are associated with immune protection against COVID-19 infection (8–10). Based on these findings, we decided to assay the levels of nAbs in serum samples of Group 1 and Group 2 at the following time points: 1 month after the second dose (T3), 7 months after the second dose (T7), and 1 month after the booster dose, merging the data of both groups (T8+T9). The automated assay we used is an inhibitory test; the higher the nAb concentration, the lower the absorbance value found (see M&M). Excluding HCWs for whom at least one of the above time points was lacking, the analysis was performed on a total of 71 HCWs (Group 1, N = 41; Group 2, N = 30), and results were expressed as a percentage of nAbs out of the total anti-spike-RBD antibodies (**Table S6**). We found that the mean percentage of nAbs was 76% at T3 and 86% at T8+T9 of the total anti-spike-RBD antibodies; and comparing T7 vs. T3 and T7 vs. T8+T9, the booster dose increased the levels of nAbs by about 3.2-fold in absolute value (**Figure 3A** and **Table S5**). This result was expected because since the nAbs are the most abundant of the total anti-spike-RBD antibodies, and since no significant differences were found between the anti-spike-RBD and nAbs at T3 (p > 0.05) nor T8+T9 (p > 0.05), the increasing levels of anti-spike-RBD should be coupled with an increase in nAbs, and *vice versa*. The result reported in **Figure 3B–D** highlights the significant correlation of the anti-spike-RBD and nAbs (r^2^ = 0.980; p < 0.001). The results at T7 (time window with the lowest anti-spike-RBD antibody titer) were unexpected because we found that the mean nAb titer dropped to 44% compared with 76% at T3 and 86% at T8+T9. So, this finding indicates that the lower the titer of anti-spike-RBD antibodies over time, the greater the loss of nAbs (p < 0.0001). Altogether, these results suggested that 1) the expression values of anti-spike-RBD and nAbs were strongly correlated, and 2) 7 months after the second dose, when the anti-spike-RBD titer was lowest, the nAb titer also decreased the most.

## Discussion

In this study, we monitored the humoral response (anti-spike-RBD and nAbs) induced by the Pfizer/BioNTech mRNA BNT162b2 vaccine in a cohort of 163 HCWs aged ≤60 years. The monitoring involved the periodic collection of peripheral blood starting from the 12th day after the first vaccine dose up to 1 month after the third vaccine dose for a total of about 1 year of surveillance. The anti-spike-RBD antibody and nAb titers were analyzed to understand their decay over time and to assess whether the humoral response after the third dose was similar to that after the second dose.

Our study has taken advantage of two cohorts that differed in the timing of administration of the third vaccine dose. Specifically, both cohorts (Group 1 and Group 2) received the first two doses in the same time window, but Group 2 received the third dose about 2 months later than Group 1 did. This dissimilarity allowed us to compare the humoral responses in the presence and absence of the third dose. Twelve days after the first vaccine dose (T1), only 86.7% of subjects showed an optimal serological response, as assessed by anti-spike-RBD antibody titer. This humoral limit was exceeded after the second vaccine dose, as 10 days after its administration (T2), 100% of subjects showed a positive anti-spike-RBD antibody response. The anti-spike-RBD antibody titers (3,366.2 BAU/ml) were highest at this time point. No difference was seen between genders, except for the time point T3 and T4, when we divided our cohort into <40 years and >40 years. In these two time points, the anti-spike-RBD antibody titer was significantly higher in women vs. men. The anti-spike-RBD antibody titer rapidly decreased from month to month until a plateau was reached 7 months after the second dose (T7). At T7, the mean value of anti-spike-RBD was 153 BAU/ml, the titer having dropped 22-fold 7 months from the second dose. Although both groups underwent the third dose differently (about 2 months apart), 100% of the subjects arrived at the third dose with a positive IgG anti-spike-RBD titer. Indeed, in the matched-pairs design, for Group 1, the mean titer of anti-spike-RBD antibodies before the third dose was 129.7 BAU/ml (T7: 7 months after the second dose), and for Group 2, it was 150.7 BAU/ml (T8*: 9 months after the second dose). Comparing T7 vs. T8*, we did not see any significant drop in the anti-spike-RBD antibody titer (p = 0.6). This result could mean that at 9 months from the second dose, the anti-spike-RBD antibody titer is almost unchanged compared with 2 months earlier. This leads us to speculate that the wane in anti-spike-RBD antibody titer slows starting from the seventh month after the second vaccine dose. This is not easy to explain, since many variables and aspects remain yet to be clarified. Group 2 received the third dose 9 months after the second dose, and our cohort lacks subjects who received the third dose after a wider time window to clarify if the wane of anti-spike-RBD antibodies effectively slows beyond 9 months from the second dose. So, further studies are needed to confirm this hypothesis.

After the third vaccine dose, in both groups, the anti-spike-RBD antibody titer rose about 34-fold (T7 vs. T8 and T8* vs. T9), and this strong increase has also been reported in other studies (22, 23). To answer the question of whether the humoral response after the third dose was similar to that after the second dose, the anti-spike-RBD antibody titer 1 month after the second dose (T3) was compared with that 1 month after the third dose (T8 and T9 for Groups 1 and 2, respectively). We found that the booster dose increased the anti-spike-RBD antibody titer 3-fold, which was in agreement with others (22–24). The changes in nAb titer over time was also assessed, as these antibodies are associated with immune protection against COVID-19 infection (10, 25). To this end, we chose three strategic time points: 1 month after the second dose (T3), 7 months after the second dose (T7), and 1 month after the third dose (T8–T9). As expected, 7 months after the second dose (T7), the nAb titer waned, and this result was consistent with previous reports (14, 15, 26–28), as was the increase in anti-spike-RBD titers (T3 and T8+T9) corresponding to a rising nAb titer (**Figure 3B**). In fact, at T3, 70% of the participants had a nAb value >91% of the total anti-spike-RBD antibody titer, which increased to 90% after the third dose (T8+T9). Interestingly, 30% of participants who showed a mean nAb titer less than 80% at T3 showed a nAb titer >91% at T8+T9. Altogether, these results showed that the booster increases the vaccine power and that the increase in anti-spike-RBD antibodies correlates with an increase in nAbs (r^2^ = 0.980; p < 0.001). On the other hand, it was unexpected to find that the nAb levels decreased more than expected at T7, i.e., when the anti-spike-RBD antibody titer reached its lowest value. Specifically, we found that, at T7, the mean nAb titer dropped to 44% compared with 76% at T3 and 86% at T8+T9. So, in this case, the lower the value of anti-spike-RBD antibodies over time, the greater the loss of nAbs (p < 0.0001). To the best of our knowledge, this is the first study finding that the ratio of anti-spike-RBD/nAbs does not remain constant but penalizes the nAbs over time. Could this excessive loss of nAbs explain why the booster dose is needed 5 or 7 months after the second dose, even though the anti-spike-RBD antibodies are still detectable? On 6 April 2022, the European Centre for Disease Prevention and Control (ECDC) and EMA’s COVID-19 task force (ETF) declared that *“it is too early to consider using a fourth dose of mRNA COVID-19 vaccines (Pfizer’s Comirnaty and Moderna’s Spikevax) in the general population”…”a fourth dose (or second booster) can be given to adults 80 years of age”…”there is currently no clear evidence in the EU that vaccine protection against severe disease is waning substantially in adults with normal immune systems aged 60 to 79 years and thus no clear evidence to support the immediate use of a fourth dose”…”If the current epidemiological situation changes and new signals emerge, it may become necessary to consider a fourth dose in this age group”* (https://www.ema.europa.eu/en/news/ecdc-ema-issue-advice-fourth-doses-mrna-covid-19-vaccines). More recent studies have focused on this question, and recent publications report studies focusing on the efficacy of a fourth dose in fragile (29, 30) and non-fragile subjects (31). Indeed, the most recent (31) involved 274 HCWs enrolled in the Sheba HCW COVID-19 cohort (5) of whom 154 received the fourth dose of BNT162b2 administered 4 months after the third dose and 120 received mRNA-1273 (Moderna) 1 week later. The authors found that the efficacy against SARS-CoV-2 infection was 30% for BNT162b2 and 11% for mRNA-1273. Nevertheless, both the controlled group (three doses) and the intervention group (four doses) reported negligible symptoms, and the maximal immunogenicity of vaccines was achieved after three doses. The authors conclude that the fourth vaccination of healthy young HCWs may have only marginal benefits.

To conclude, the longevity of the humoral immunity after infection and vaccination is still an ongoing debate, and a threshold of the nAb titer related to protective activity remains to be defined. However, we cannot overlook the fact that millions of citizens received three administrations of the same vaccine in less than 1 year, and yet they continue to be infected. So, we wonder: Why is this so? If the virus has changed its genetics, but the BNT162b2 vaccine is still the same, is it plausible to suspect that the number of infections does not decrease because the vaccine does not prevent infection with the new virus variants? Maybe this is why the Pfizer company is working to produce a vaccine that works against not only the dominant Omicron but all known COVID-19 variants. In the absence of any other technical and scientific explanation, should we recommend a vaccine booster every 5 or 6 months, and for how long? In addition, might there be any immune consequences of the periodic administration of the same vaccine over a short period? No vaccine is completely free from risk, and among the adverse reactions to vaccines, one of the most feared is the triggering of autoimmune diseases (32, 33). We cannot answer these questions with our limited study: the study cohort is relatively small, the protective role of nAbs has not been evaluated, and the B lymphocyte immunity has not been examined in this report, although it is ongoing in another multicenter study in which we are a partner. In addition, although certified as a CE-IVD test and applied in clinical practice, the nAb kit used is a surrogate virus neutralization test. We believe that our data can improve knowledge related to humoral response following vaccination against COVID-19 infection, enriching recent literature data (34–37). Certainly, a comprehensive assessment is needed to manage this infection. We believe it would be appropriate for those research groups studying huge cohorts and publishing epidemiological and clinical trial results to design studies to examine the relationship between nAbs and anti-spike-RBD antibody levels (thresholds) and their efficacy against symptomatic SARS-CoV-2 infection.

## Data availability statement

The original contributions presented in the study are included in the article/**Supplementary Material**. Further inquiries can be directed to the corresponding author.

## Ethics statement

This study was reviewed and approved by Ethic Committee of the Istituto Nazionale Tumori IRCCS Fondazione G. Pascale (Protocollo 4/21SDN). The patients/participants provided their written informed consent to participate in this study.

## Author contributions

Conceptualization: MI, CO, MS; samples collection: LCo; investigation: MI, RS, SS, LCi; data analysis: MF, MI; writing and original draft preparation: MI, MF; writing, review, and editing: LCo, CO, LCi, SS; funding acquisition: CO, MI, and MS. All authors read and approved the final version of the manuscript.

## Funding

This research was funded by Ministero della Salute on the Ricerca Corrente funds RRC-2022-23680785.

## Acknowledgments

I would like to thank all my colleagues who decided to participate in this study. A special thanks to my colleague Anna Maria Grimaldi, who supported me when needed. I want to thank the BBMRI (Biobanking and BioMolecular resources Research Infrastructure) network of which SDN Biobank is a part.

## Conflict of interest

Author SS is employed by Synlab Lazio Srl. 

The remaining authors declare that the research was conducted in the absence of any commercial or financial relationships that could be construed as a potential conflict of interest.

## Publisher’s note

All claims expressed in this article are solely those of the authors and do not necessarily represent those of their affiliated organizations, or those of the publisher, the editors and the reviewers. Any product that may be evaluated in this article, or claim that may be made by its manufacturer, is not guaranteed or endorsed by the publisher.

## References

[1] Polack FP Thomas SJ Kitchin N Absalon J Gurtman A Lockhart S. Safety and efficacy of the BNT162b2 mRNA covid-19 vaccine. N Engl J Med (2020) 383:2603–15. doi: 10.1056/NEJMoa2034577 PMC7745181 33301246

[2] Walsh EE Frenck RW Falsey AR Kitchin N Absalon J Gurtman A. Safety and immunogenicity of two RNA-based covid-19 vaccine candidates. N Engl J Med (2020) 383:2439–50. doi: 10.1056/NEJMoa2027906 PMC7583697 33053279

[3] Haas EJ Angulo FJ McLaughlin JM Anis E Singer SR Khan F. Impact and effectiveness of mRNA BNT162b2 vaccine against SARS-CoV-2 infections and COVID-19 cases, hospitalisations, and deaths following a nationwide vaccination campaign in Israel: An observational study using national surveillance data. Lancet (2021) 397:1819–29. doi: 10.1016/s0140-6736(21)00947-8 PMC8099315 33964222

[4] Goldberg Y Mandel M Bar-On YM Bodenheimer O Freedman L Haas EJ. Waning immunity after the BNT162b2 vaccine in Israel. N Engl J Med (2021) 385(24):1–10. doi: 10.1056/NEJMoa2114228 34706170 PMC8609604

[5] Levin EG Lustig Y Cohen C Fluss R Indenbaum V Amit S. Waning immune humoral response to BNT162b2 covid-19 vaccine over 6 months. N Engl J Med (2021) 385(24):1–11. doi: 10.1056/NEJMoa2114583 34614326 PMC8522797

[6] Bar-On YM Goldberg Y Mandel M Bodenheimer O Freedman L Kalkstein N. Protection of BNT162b2 vaccine booster against covid-19 in Israel. N Engl J Med (2022) 385(15):1393–400. doi: 10.1056/NEJMoa2114255 PMC8461568 34525275

[7] Ben David SS Shamir-Stein N Gez SB Lerner U Rahamim-Cohen D Zohar AE. Reactogenicity of a third BNT162b2 mRNA COVID-19 vaccine among immunocompromised individuals and seniors-A nationwide survey. Clin Immunol (2021) 232:1–4. doi: 10.1016/j.clim.2021.108860 PMC8461972 34571262

[8] Garcia-Beltran WF Lam EC Astudillo MG Yang DN Miller TE Feldman J. COVID-19-neutralizing antibodies predict disease severity and survival. Cell (2021) 184:476–+. doi: 10.1016/j.cell.2020.12.015 PMC7837114 33412089

[9] Yang Y Du LY. SARS-CoV-2 spike protein: A key target for eliciting persistent neutralizing antibodies. Signal Transduct Targeted Ther (2021) 6:1–3. doi: 10.1038/s41392-021-00523-5 PMC7908000 33637679

[10] Khoury DS Cromer D Reynaldi A Schlub TE Wheatley AK Juno JA. Neutralizing antibody levels are highly predictive of immune protection from symptomatic SARS-CoV-2 infection. Nat Med (2021) 27:1205–+. doi: 10.1038/s41591-021-01377-8 34002089

[11] Wu F Liu M Wang AJ Lu L Wang QM Gu CJ. Evaluating the association of clinical characteristics with neutralizing antibody levels in patients who have recovered from mild COVID-19 in shanghai, China. JAMA Internal Med (2020) 180:1356–62. doi: 10.1001/jamainternmed.2020.4616 PMC9377417 32808970

[12] Wang XL Guo XH Xin QQ Xin Y Pan YL Hu J. Neutralizing antibody responses to severe acute respiratory syndrome coronavirus 2 in coronavirus disease 2019 inpatients and convalescent patients. Clin Infect Dis (2020) 71:2688–94. doi: 10.1093/cid/ciaa721 PMC7314147 32497196

[13] Dispinseri S Secchi M Pirillo MF Tolazzi M Borghi M Brigatt C. Neutralizing antibody responses to SARS-CoV-2 in symptomatic COVID-19 is persistent and critical for survival. Nat Commun (2021) 12:1–12. doi: 10.1038/s41467-021-22958-8 33976165 PMC8113594

[14] Terpos E Trougakos IP Karalis V Ntanasis-Stathopoulos I Gumeni S Apostolakou F. Kinetics of anti-SARS-CoV-2 antibody responses 3 months post complete vaccination with BNT162b2; a prospective study in 283 health workers. Cells (2021) 10:1–17. doi: 10.3390/cells10081942 PMC8394923 34440710

[15] Terpos E Karalis V Ntanasis-Stathopoulos I Gavriatopoulou M Gumeni S Malandrakis P. Robust neutralizing antibody responses 6 months post vaccination with BNT162b2: A prospective study in 308 healthy individuals. Life-Basel (2021) 11:1–9. doi: 10.3390/life11101077 PMC8537997 34685448

[16] Malipiero G D’Agaro P Segat L Moratto A Villalta D. Long-term decay of anti-RBD IgG titers after BNT162b2 vaccination is not mirrored by loss of neutralizing bioactivity against SARS-CoV-2. Clin Chim Acta (2022) 524:11–7. doi: 10.1016/j.cca.2021.11.023 PMC8630423 34843705

[17] Eliakim-Raz N Leibovici-Weisman Y Stemmer A Ness A Awwad M Ghantous N. Antibody titers before and after a third dose of the SARS-CoV-2 BNT162b2 vaccine in adults aged >= 60 years. Jama-J Am Med Assoc (2021) 326(21):2203–04. doi: 10.1001/jama.2021.19885 PMC8652594 34739043

[18] Mirabelli P Incoronato M Coppola L Infante T Parente CA Nicolai E. SDN biobank: Bioresource of human samples associated with functional and/or morphological bioimaging results for the study of oncological, cardiological, neurological, and metabolic diseases. Open J Bioresour (2017) 4:1–4. doi: 10.5334/ojb.26

[19] Albano PM Notarte KI Macaranas I Maralit B. Cross-contamination in molecular diagnostic laboratories in low-and middle-income countries. PJP (2020) 5:7–11. doi: 10.21141/PJP.2020.09

[20] Notarte KI Ver AT Velasco JV Pastrana A Catahay JA Salvagno GL. Effects of age, sex, serostatus, and underlying comorbidities on humoral response post-SARS-CoV-2 pfizer-BioNTech mRNA vaccination: A systematic review. Crit Rev Clin Lab Sci (2021) 59(6):373–90. doi: 10.1080/10408363.2022.2038539 PMC8935447 35220860

[21] Notarte KI Guerrero-Arguero I Velasco JV Ver MH AT de Oliveira S Catahay JA. Characterization of the significant decline in humoral immune response six months post-SARS-CoV-2 mRNA vaccination: A systematic review. J Med Virol (2022) 94:2939–61. doi: 10.1002/jmv.27688 PMC9088566 35229324

[22] Busa R Sorrentino MC Russelli G Amico G Miceli V Miele M. Specific anti-SARS-CoV-2 humoral and cellular immune responses after booster dose of BNT162b2 pfizer-BioNTech mRNA-based vaccine: Integrated study of adaptive immune system components. Front Immunol (2022) 13:856657. doi: 10.3389/fimmu.2022.856657 35401503 PMC8987231

[23] Lau CS Phua SK Liang YL Oh MLH Aw TC. SARS-CoV-2 spike and neutralizing antibody kinetics 90 days after three doses of BNT162b2 mRNA COVID-19 vaccine in Singapore. Vaccines (2022) 10:1–8. doi: 10.3390/vaccines10020331 PMC8879250 35214789

[24] Stasi C Meoni B Voller F Silvestri C. SARS-CoV-2 vaccination and the bridge between first and fourth dose: Where are we? Vaccines (2022) 10:1–21. doi: 10.3390/vaccines10030444 PMC8953092 35335075

[25] Earle KA Ambrosino DM Fiore-Gartland A Goldblatt D Gilbert PB Siber GR. Evidence for antibody as a protective correlate for COVID-19 vaccines. Vaccine (2021) 39:4423–8. doi: 10.1016/j.vaccine.2021.05.063 PMC8142841 34210573

[26] Hu C Li D Liu Z Ren L Su J Zhu M. Exploring rapid and effective screening methods for anti-SARS-CoV-2 neutralizing antibodies in COVID-19 convalescent patients and longitudinal vaccinated populations. Pathog (Basel Switzerland) (2022) 11:1–12. doi: 10.3390/pathogens11020171 PMC8878712 35215115

[27] Terpos E Karalis V Ntanasis-Stathopoulos I Evangelakou Z Gavriatopoulou M Manola MS. Comparison of neutralizing antibody responses at 6 months post vaccination with BNT162b2 and AZD1222. Biomedicines (2022) 10. doi: 10.3390/biomedicines10020338 PMC8961789 35203547

[28] Infantino M Manfredi M Stacchini L Cosma C Grossi V Lari B. The role of neutralizing antibodies by sVNT after two doses of BNT162b2 mRNA vaccine in a cohort of Italian healthcare workers. Clin Chem Lab Med (2022) 60(6):934–40. doi: 10.1515/cclm-2022-0170 35303766

[29] Benotmane I Bruel T Planas D Fafi-Kremer S Schwartz O Caillard S. A fourth dose of the mRNA-1273 SARS-CoV-2 vaccine improves serum neutralization against the delta variant in kidney transplant recipients. Kidney Int (2022) 101(5):1073–76. doi: 10.1016/j.kint.2022.02.011 PMC8881812 35231463

[30] Housset P Kubab S Hanafi L Pardon A Vittoz N Bozman D-F. Humoral response after a fourth “booster” dose of a covid-19 vaccine following a 3-dose regimen of mRNA-based vaccination in dialysis patients. Kidney Int (2022) 101(6):1289–90. doi: 10.1016/j.kint.2022.04.006 PMC9001011 35421509

[31] Regev-Yochay G Gonen T Gilboa M Mandelboim M Indenbaum V Amit S. Efficacy of a fourth dose of covid-19 mRNA vaccine against omicron. N Engl J Med (2022) 386:1377–80. doi: 10.1056/NEJMc2202542 PMC9006792 35297591

[32] Olivieri B Betterle C Zanoni G. Vaccinations and autoimmune diseases. Vaccines (2021) 9:1–15. doi: 10.3390/vaccines9080815 PMC8402446 34451940

[33] Bouattour N Hdiji O Sakka S Fakhfakh E Moalla K Daoud S. Guillain-Barre syndrome following the first dose of pfizer-BioNTech COVID-19 vaccine: Case report and review of reported cases. Neurol Sci (2022) 43:755–61. doi: 10.1007/s10072-021-05733-x PMC8601771 34796417

[34] Niyomnaitham S Toh ZQ Wongprompitak P Jansarikit L Srisutthisamphan K Sapsutthipas S. Immunogenicity and reactogenicity against the SARS-CoV-2 variants following heterologous primary series involving CoronaVac, ChAdox1 nCov-19 and BNT162b2 plus BNT162b2 booster vaccination: An open-label randomized study in healthy Thai adults. Hum Vaccines Immunotherapeut. doi: 10.1080/21645515.2022.2091865 PMC9746495 35816053

[35] Herzberg J Fischer B Becher H Becker AK Honarpisheh H Guraya SY. Short-term drop in antibody titer after the third dose of SARS-CoV-2 BNT162b2 vaccine in adults. Vaccines (2022) 10:1–6. doi: 10.3390/vaccines10050805 PMC9145913 35632564

[36] Padoan A Cosma C della Rocca F Barbaro F Santarossa C Dall'Olmo L. A cohort analysis of SARS-CoV-2 anti-spike protein receptor binding domain (RBD) IgG levels and neutralizing antibodies in fully vaccinated healthcare workers. Clin Chem Lab Med (2022) 60:1110–5. doi: 10.1515/cclm-2022-0322 35473824

[37] Seki Y Yoshihara Y Nojima K Momose H Fukushi S Moriyama S. Safety and immunogenicity of the Pfizer/BioNTech SARS-CoV-2 mRNA third booster vaccine dose the BA.1 and BA.2 omicron variants. Med (2022) 3:406–+. doi: 10.1016/j.medj.2022.04.013 PMC9040508 35815933

