# Supplementary figures and images for: SARS-CoV-2 antibody responses before and after a third dose of the BNT162b2 vaccine in Italian healthcare workers aged ≤60 years: One year of surveillance
Source: Front Immunol. 2022 Sep 30;13:947187. doi: 10.3389/fimmu.2022.947187 (PMC9566572; doi:10.3389/fimmu.2022.947187)

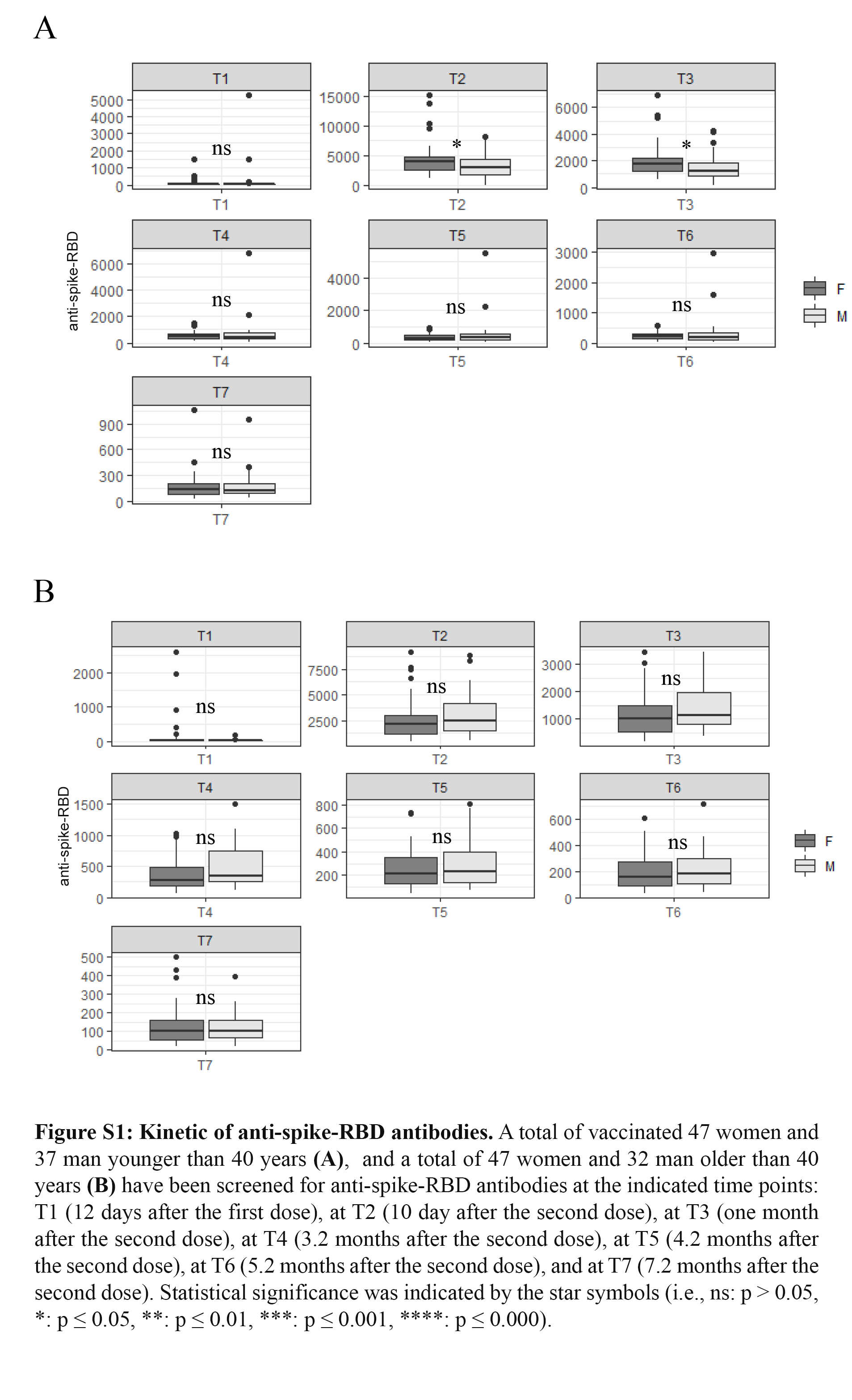

Supplement: Supplementary file 7 [file Image_1.tif]
